# LRP11-AS1 promotes the proliferation and migration of triple negative breast cancer cells via the miR-149-3p/NRP2 axis

**DOI:** 10.1186/s12935-022-02536-8

**Published:** 2022-03-12

**Authors:** Peng Li, Yu Zeng, Yudan Chen, Peng Huang, Xinchun Chen, Weidong Zheng

**Affiliations:** 1 Department of Laboratory Medicine, Shenzhen University General Hospital, No. 1098 Xueyuan Ave, Nanshan District, Shenzhen, 518055 China; 2 Marshall Laboratory of Biomedical Engineering, International Cancer Center, School of Biomedical Engineering, Shenzhen University Health Science Center, Shenzhen, China; 3 Guangdong Provincial Key Laboratory of Regional Immunity and Diseases, Health Science Center, Shenzhen University, Shenzhen, China

**Keywords:** LRP11-AS1, lncRNA, miR-149-3p, NRP2, Breast cancer

## Abstract

**Background:**

Breast cancer is the most commonly diagnosed cancer in women. Triple negative breast cancer (TNBC) is the most difficult subtype of breast cancer to treat due to the deficiency in drug-targetable receptors. LRP11-AS1, a newly identified oncogenic long noncoding RNA (lncRNA) was found to be significantly overexpressed in TNBC cells. The aim of this study is to investigate the malignant roles and the oncogenic mechanisms of LRP11-AS1 in TNBC.

**Methods:**

CCK-8, colony formation, transwell migration and transwell invasion assays were performed to study the functions of LRP11-AS1. Quantitative PCR and western blot were used to determine the gene expression. Bioinformatics analysis and dual-luciferase reporter assay were conducted to study lncRNA and miRNA interactions.

**Results:**

LRP11-AS1 was found to be significantly overexpressed in TNBC cells compared to the non-TNBC cells and normal mammary epithelial cells. Knockdown of LRP11-AS1 could inhibit the growth and metastasis of TNBC cells and regulate cell cycle. Mechanistically, LRP11-AS1 was found to act as a competing endogenous RNA (ceRNA) to sponge miR-149-3p. Silencing of LRP11-AS1 increased the expression of miR-149-3p and overexpression of miR-149-3p suppressed the expression of LRP11-AS1. Inhibition of miR-149-3p could reverse the anticancer effect of LRP11-AS1 deficiency in TNBC cells. Moreover, Neuropilin-2 (NRP2) was found to be the target of miR-149-3p. Rescue experiments revealed that NRP2 overexpression could rescue the anticancer effect of LRP11-AS1 deficiency in TNBC cells.

**Conclusion:**

LRP11-AS1 overexpressed in TNBC showed the oncogenic effects possibly by sponging miR-149-3p and regulating the miR-149-3p/NRP2 axis, which indicated LRP11-AS1 as a potential diagnostic biomarker and therapeutic target in TNBC.

**Supplementary Information:**

The online version contains supplementary material available at 10.1186/s12935-022-02536-8.

## Background

Breast cancer is the most frequently diagnosed cancer in women. In 2020, female breast cancer has surpassed lung cancer as the most commonly diagnosed cancer, with an estimation of 2.3 million new cases, leading to the death of 680,000 patients worldwide [1]. Treatment of breast cancer is a multidisciplinary management, including surgery, radiation and medical therapy. Medical treatment such as chemotherapy, endocrine therapy or HER-2 directed therapy is determined by factors including disease stage, hormone receptor status, lymph node involvement and tumor size [2]. Triple negative breast cancer (TNBC) is a subtype of breast cancer defined by a deficiency in estrogen, progesterone and HER2 receptors. TNBC was highly aggressive with high recurrence and organ metastasis rate. Due to the lack of drug-targetable receptors, chemotherapy is the only recommended systemic treatment of TNBC, inevitably with resistance and relapse. New diagnostic biomarkers and drug targets are needed for the treatment of this type of breast cancer [3, 4].

LncRNAs are long noncoding RNAs with more than 200 nucleotides, functioning as master regulators of gene expression in various biological events and disease processes including cancer. They could regulate the targeted genes in the transcriptional or post-transcriptional levels [5]. A recent “competing endogenous RNA (ceRNA)” hypothesis proposed that lncRNA could regulate gene expression by sequestering microRNAs (miRNAs) to control their concentration within the cell and act as a negative regulator of miRNAs and therefore, a positive regulator of the targeted gene expression [6, 7]. ENSG00000273132, located at chromosome 6 is a novel long noncoding RNA (lncRNA) antisense to low-density lipoprotein (LDL) receptor related protein 11 (LRP11). We here named it LRP11 antisense RNA 1 (LRP11-AS1). This lncRNA was reported to be overexpressed in papillary thyroid cancer [8]. However, to date, the expression of LRP11-AS1 in other cancer apart from thyroid cancer has not been investigated. We analyzed the expression of LRP11-AS1 in the cell lines of different cancer types. Interestingly, we found that LRP11-AS1 was significantly overexpressed in TNBC cells as compared to the non-TNBC cells and the normal mammary epithelial cells. The roles of LRP11-AS1 played in TNBC and the possible mechanisms of its oncogenic behavior are therefore investigated here.

On the basis of target prediction using bioinformatics analyses, LRP11-AS1 may serve as a ceRNA for miR-149-3p. miR-149-3p was reported to be a tumor suppressive miRNA in breast cancer. The expression of miR-149-3p was reported to be decreased in the clinical samples of breast cancer. Inhibition of miR-149-3p could promote the proliferation, invasion and migration of breast cancer cells [9, 10]. Moreover, online bioinformatics analysis tool TargetScan (http://www.targetscan.org/vert_72/) predicted Neuropilin-2 (NRP2) as one potential target of miR-149-3p. NRP2 is a member of neuropilin family, which are cell surface antigens that bind to vascular endothelial growth factor (VEGF). NRP2 was found to be involved in multiple cellular physiological functions including proliferation, angiogenesis and migration [11–13]. It was found to be overexpressed in invasive breast cancer and TNBC, and was correlated with lymph node metastasis [12, 14].

In this work, we explored the relationships among LRP11-AS1, miR-149-3p and NRP2 in triple negative breast cancer cells. Our findings may provide a potential diagnostic biomarker and therapeutic target in TNBC.

## Materials and methods

### Cell culture and transfection

MDA-MB-231, MDA-MB-468, MCF7 and MCF10A cells were purchased from cell bank of Chinese Academy of Sciences (Shanghai, China). Cells were cultured in RPMI 1640 (8121465, Gibco, USA) with 10% Foetal Bovine Serum (FBS, 1709A, Bovogen, Australia) and 1% penicillin–streptomycin (25200–056, Gibco, USA) at 37 °C in an incubator in a saturated humidified atmosphere supplied with 5% CO_2_. MCF10A was cultured in the same cell culture conditions but with 20% FBS. Knockdown of LRP11-AS1 was performed by transiently transfected the cells with smart silencer (RIBOBIO, China) using lipofectamine 2000 (2067563, Invitrogen, USA) according to the manufacturer’s protocol. Smart silencer is a mixture of 3 siRNAs and 3 antisense oligonucleotides. The targeted sequences of siRNAs were 5′-CCAGCAGACCTAGCTGCCA-3′, 5′-CTAGCTGCCAGCAGTGACA-3′, 5′-CATCCTTTTCTGAGAAAGA-3′. The targeted sequences of antisense oligonucleotides were 5′-GACAACATGCGTTACCTTCA-3′, 5′-TGCAGCAGTGCCCACTCATA-3′, 5′-TGGTCATCTGTGCTCTCGCG-3′. Overexpression of LRP11-AS1 was conducted using lentivirus. The virus were packed with packaging plasmid psPAX2, pMD2.G and PHBLVTM, and recombinant plasmid pHBLV-CMV-MCS-EF1-ZsGreen-T2A-Puro encoding the sequence of LRP11-AS1 (ENSG00000273132, Hanbio Biotechnology, China). NRP2 overexpression plasmid (HG10695-CF) and control plasmid (CV012) were purchased from Sino Biological (Beijing, China). miRNA mimics and inhibitors were purchased from RIBOBIO (Guangzhou, China). Cells were transfected using lipofectamine 2000 (2067563, Invitrogen, USA) according to the manufacturer’s instructions.

### Cell proliferation and colony formation assay

A total of 5000 cells/well in 100 μL of culture media were seeded in the 96-well plate. CCK-8 reagent (HB-CCK8-1, Hanbio Biotechnology, China) was used to determine cell proliferation according to the manufacturer’s instructions. OD450 was determined at different time points (day 0, 1, 2,3 and 4 for the knockdown study and day 0, 1, 2 and 3 for the overexpression study) after CCK-8 incubation. Colony formation was performed by seeding cells in 6-well plate and allowed to grow for 8 days followed by fixing with methanol and staining with crystal violet. The cell number seeded in the well was as follows: A number of 5000 cells/well of MDA-MB-468 cells were seeded. A number of 3000 cells/well of MDA-MB-231 cells were seeded except for the LRP11-AS1 overexpression experiment, 2000 cells/well were seeded. The media used in the colony formation assay were 2 mL/well.

### Soft agar colony formation assay

The base agar layer consisted of 0.5% of low-gelling-point agarose (C12595402, Macklin, China) in culture medium. Top agar layer consisting of 0.3% of low-gelling-point agarose in culture medium with 6000/well of transfected MDA-MB-231 or MDA-MB-468 cells were plated on top of the base agar in 6-well plate. Cells were allowed to grow for 14 days before taking photos under microscope.

### Migration and invasion assay

For cell migration assay, 5 × 10^4 cells/well of MDA-MB-231 or 1 × 10^5 cells/well of MDA-MB-468 cells were seeded into the upper chamber of the transwell insert (725301, NEST) in RPMI medium without FBS. The lower chamber was added with RPMI medium with 10% FBS. Cells were allowed to migrate for 24 h. After that, the cells in the upper chamber of the insert were removed and the migrated cells at the bottom of the insert membrane were fixed with methanol and stained with crystal violet. For cell invasion assay, the membrane of the transwell insert was coated with Matrigel (354234, Corning,) according to the manufacturer’s instructions. A total of 8 × 10^4 cells/well of MDA-MB-231 cells were seeded in the upper chamber of the insert. Other conditions were the same as cell migration assay.

### Wound healing assay

A total of 4 × 10^5 cells/well of transfected MDA-MB-231 cells or a total of 6 × 10^5 cells/well of transfected MDA-MB-468 cells were seeded in 12-well plate for 24 h. Wound was scratched using a 100μL tip. The cells were allowed to migrate for 24 h. Images were taken at 0 h and 24 h after wound scratching.

### Western blot

Cell lysate was prepared with RIPA lysis buffer supplemented with 1 × protease inhibitor (70090050, Biosharp, China) and 1 × phosphatase inhibitor (70080020, Biosharp, China) at 4 °C. Western blot was performed according to the common protocol. The primary antibodies used were anti-cyclin D1 antibody (92G2, Cell Signaling Technology), anti-NRP2 antibody (D39A5, Cell Signaling Technology), anti-p21 antibody (12D1, Cell Signaling Technology) and anti-GAPDH (D16H11, Cell Signaling Technology) with a dilution of 1:1000. Anti-rabbit secondary antibody (7074S, Cell Signaling Technology) was used with a dilution of 1:3000.

### RNA extraction, reverse transcription and quantitative PCR (qPCR)

Extraction of total RNA and miRNA was conducted using RNA extraction kit (DP419, DP501, Tiangen, China). Reverse transcription of total RNA and miRNA was conducted using reverse transcription kits (RR037A, Takara and KR211, Tiangen). qPCR was performed using LightCycler 480 instrument (Roche) and SYBR green qPCR kit (AG11701, Accurate Biology, China). The primers used in the qPCR assays were: LRP11-AS1 forward: 5′-CTAGCTGCCAGCAGTGACAA-3′, LRP11-AS1 reverse: 5′-GCGAGAGCACAGATGACCAC-3′; β-actin forward: 5′-TGGCATCCACGAAACTACCT-3′, β-actin reverse: 5′-ACGGAGTACTTGCGCTCAG-3′; NRP2 forward: 5′-GTGGACCTGCGCTTTTTAACC-3′, NRP2 reverse: 5′-GCCATTCTGTGTTTCCCTGG-3′. The forward and reversed primers for the quantitation of miR-149-3p was designed and synthesized by Tiangen. The primers for the quantitation of U6 were: U6 forward: 5′-AACGCTTCACGAATTTGCGT-3′, U6 reverse: 5′-CTCGCTTCGGCAGCACA-3′.

### Flow cytometry

A total of 1 × 10^5 cells/well were seeded in 12-well plate for 24 h before transfection with smart silencer for 72 h followed by cell cycle analysis using cell cycle analysis kit from BD (550825). BD FACSCanto II flow cytometry was used as assay system.

### Dual-luciferase reporter assay

Fragments of LRP11-AS1 containing the binding site of miR-149-3p or the mutant sequence were synthesized and sub-cloned into the pmirGLO dual-luciferase reporter plasmid (GENEWIZ, China). Cells were seeded in 24-well plate at a density of 5 × 10^4 cells/well for 24 h before co-transfection with miR-149-3p mimics or mimics NC and the wild type dual-luciferase reporter plasmid or the mutant plasmid. Luciferase activity was determined at 48 h post-transfection using the Dual-Luciferase Reporter Assay System (E1910, Promega). Fragments of the 3′-untranslated region (3′’-UTR) of NRP2 containing the binding site of miR-149-3p or the mutant sequence were synthesized and sub-cloned into the pmirGLO dual-luciferase reporter plasmid (GENEWIZ, China). Other experiment conditions were the same.

### Statistical analysis

All quantitative results were presented as the mean ± standard deviation (SD). The Student’s t-test (two-sided) was used to compare the statistical significance between two groups using Prism. Differences were considered significant as *P < 0.05; **P < 0.01, ***P < 0.001.

## Results

### LRP11-AS1 promoted the growth and metastasis of TNBC cells and regulated cell cycle

**Fig. 1:**
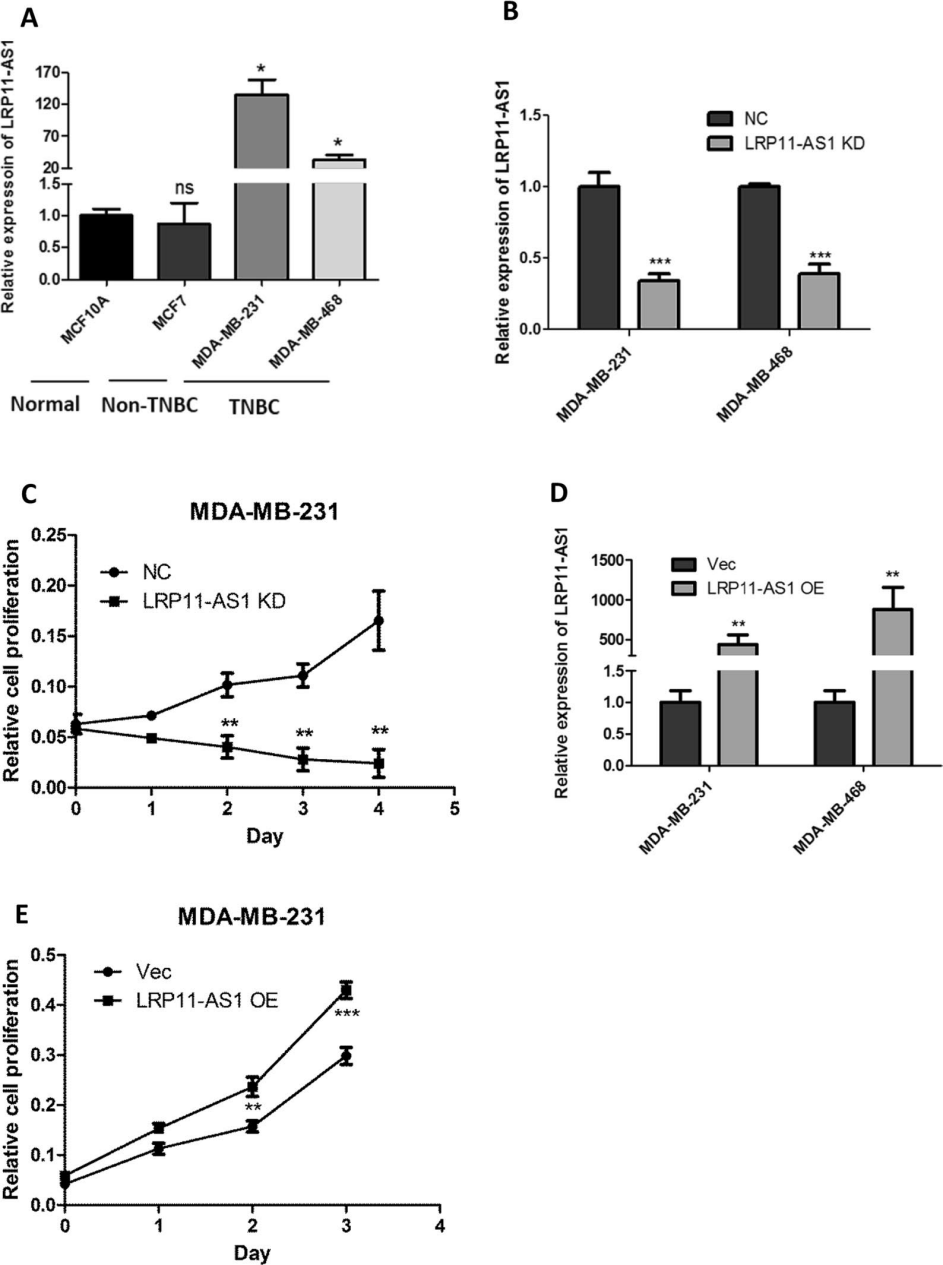

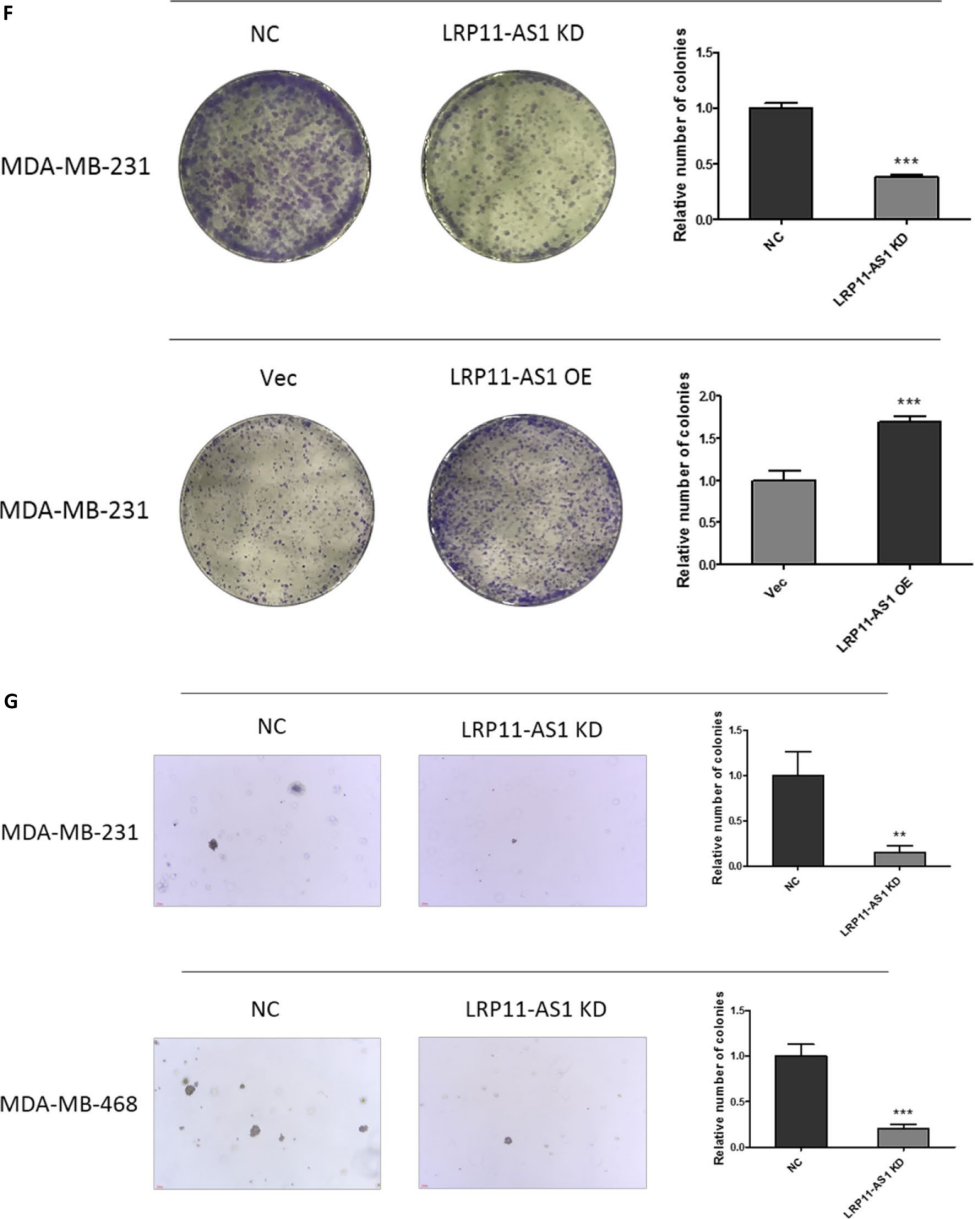

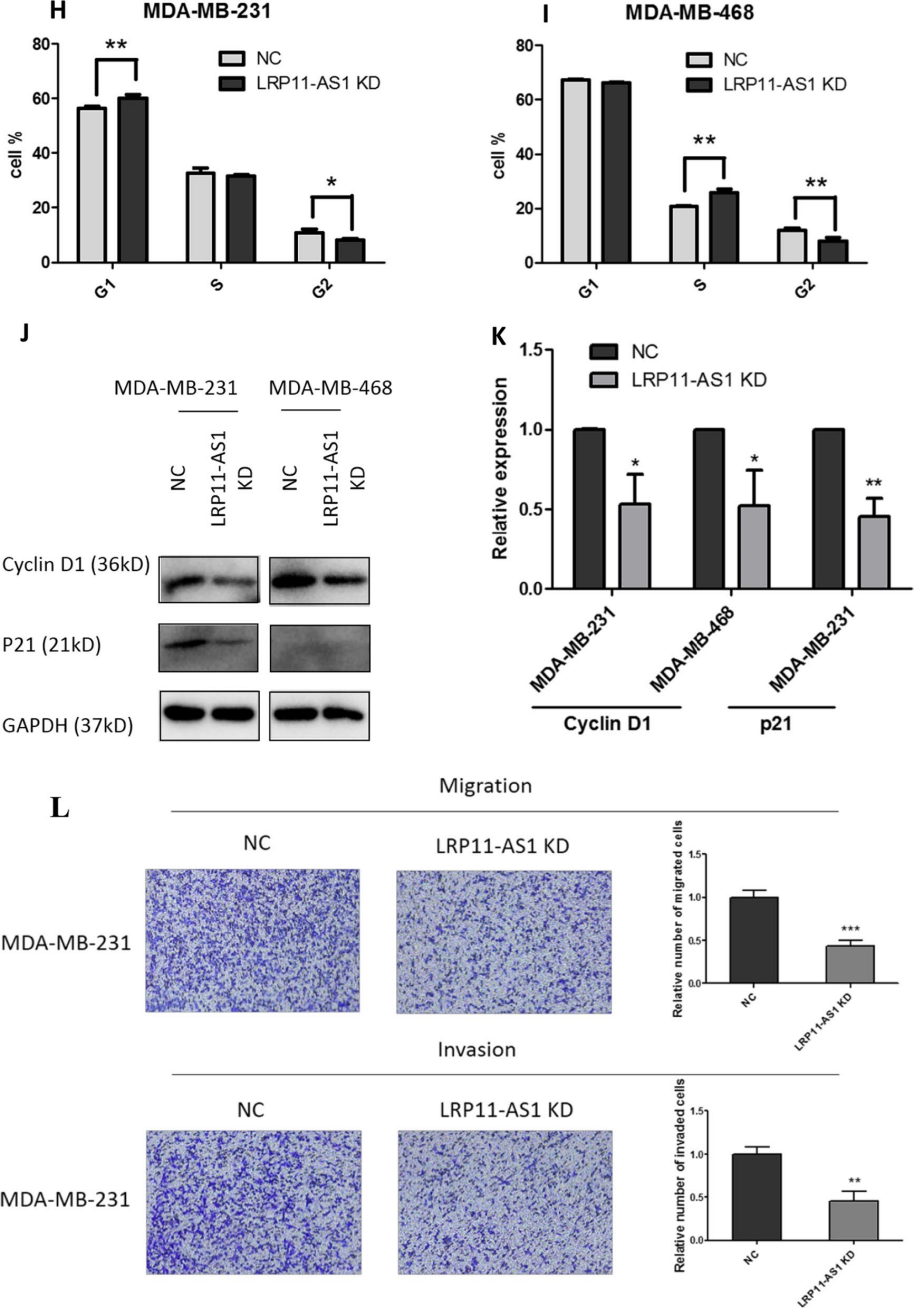

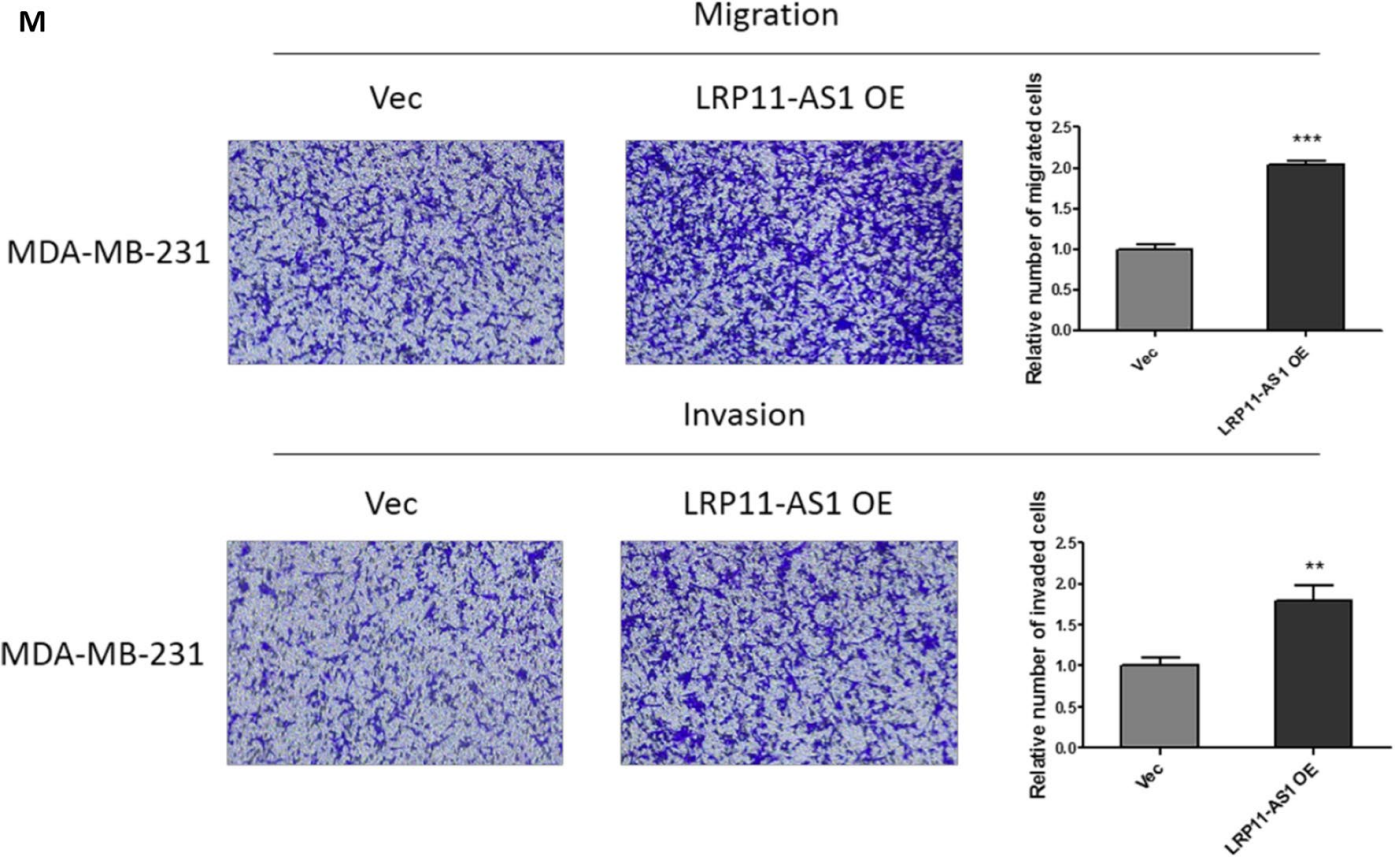
LRP11-AS1 promoted the growth and metastasis of TNBC cells and regulated cell cycle. **A** LRP11-AS1 was found to be overexpressed in TNBC cells. The expression of LRP11-AS1 was evaluated by qPCR. **B** LRP11-AS1 was knocked down in TNBC cells. The expression of LRP11-AS1 was evaluated by qPCR. **C** Knockdown of LRP11-AS1 inhibited the proliferation of TNBC cells. CCK-8 assay was performed to determine the proliferation of TNBC cells. **D** LRP11-AS1 was overexpressed in TNBC cells. The expression of LRP11-AS1 was evaluated by qPCR. **E** Overexpression of LRP11-AS1 promoted the proliferation of TNBC cells. **F** Colony formation assay of TNBC cells knocked down with LRP11-AS1 or overexpressed with LRP11-AS1. **G** Knockdown of LRP11-AS1 inhibited the soft agar colony formation of TNBC cells. Bar = 100 μm, the images were taken under 4X magnification. **H**, **I** Knockdown of LRP11-AS1 induced cell cycle arrest of TNBC cells. **J**, **K** Knockdown of LRP11-AS1 downregulated cell cycle checkpoint cyclin D1 and p21. Western blot results were shown in **J** and quantitation of western blots was shown in **K**. **L** Silencing of LRP11-AS1 inhibited the migration and invasion of TNBC cells. The images were taken under ×4 magnification. **M** Overexpression of LRP11-AS1 enhanced the migration and invasion of TNBC cells. The images were taken under ×4 magnification. Data were presented as mean ± SD. Statistic significant differences were indicated as *P < 0.05, **P < 0.01, ***P < 0.001

In our previous research, LRP11-AS1 was found to be an oncogenic lncRNA in thyroid cancer. The role of LRP11-AS1 in breast cancer was investigated here. As shown in Fig. 1A, LRP11-AS1 was found to be overexpressed in TNBC cell line MDA-MB-231 and MDA-MB-468 compared to the non-TNBC cell line MCF7 and the mammary epithelial cell line MCF10A. Knockdown of LRP11-AS1 (Fig. 1B) inhibited the proliferation of TNBC cells (Fig. 1C and Additional file 1: Fig. S1). Overexpression of LRP11-AS1 (Fig. 1D) promoted the proliferation of TNBC cells (Fig. 1E and Additional file 1: Fig. S2). Silencing of LRP11-AS1 inhibited whereas overexpression promoted the colony formation of TNBC cells (Fig. 1F and Additional file 1: Fig. S3). Silencing of LRP11-AS1 also inhibited the soft agar colony formation of TNBC cells (Fig. 1G). Cell cycle analysis indicated that silencing of LRP11-AS1 inhibited cell cycle G1/S transition of MDA-MB-231 cells (Fig. 1H) and inhibited cell cycle S/G2 transition of MDA-MB-468 cells (Fig. 1I). The expression of cyclin D1 was downregulated after the knockdown of LRP11-AS1 in both MDA-MB-231 and MDA-MB-468 cells. The expression of p21 was suppressed after the knockdown of LRP11-AS1 in MDA-MB-231. The basal expression of p21 in MDA-MB-468 was low (Fig. 1J and 1K). The effect of LRP11-AS1 on the metastasis of TNBC cells was evaluated. Knockdown of LRP11-AS1 inhibited the migration and invasion of TNBC cells (Fig. 1L and Additional file 1: Fig. S4). Overexpression of LRP11-AS1 promoted the migration and invasion of TNBC cells (Fig. 1M and Additional file 1: Fig. S5). Knockdown of LRP11-AS1 also inhibited the wound healing migration of TNBC cells (Additional file 1: Fig. S6). These results suggested that LRP11-AS1 could promote the growth and migration of TNBC cells and also regulate cell cycle.

### miR-149-3p was the target of LRP11-AS1 in TNBC

**Fig. 2:**
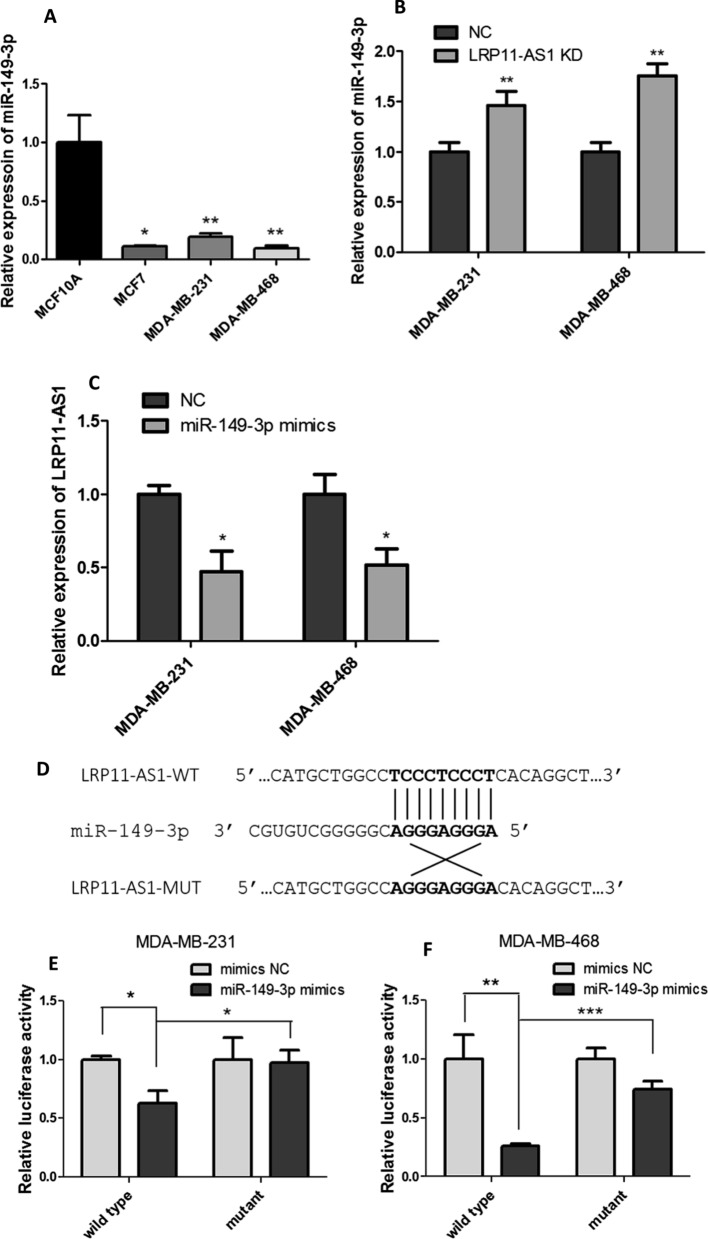
miR-149-3p was the target of LRP11-AS1 in TNBC. **A** The expression of miR-149-3p was found to be downregulated in TNBC cells and negatively correlated to LRP11-AS1. The expression of miR-149-3p was evaluated by qPCR. **B** Knockdown of LRP11-AS1 increased the expression of miR-149-3p in TNBC cells. **C** Overexpression of miR-149-3p decreased the expression of LRP11-AS1 in TNBC cells. **D** The predicted binding sequence between LRP11-AS1 and miR-149-3p. **E**, **F** Dual-luciferase reporter assay of the interaction between LRP11-AS1 and miR-149-3p. Cells were co-transfected with miRNA mimics and the dual-luciferase reporter plasmid. Luciferase activity was measured at 48 h post-transfection. Data were presented as mean ± SD. Statistic significant differences were indicated as *P < 0.05, **P < 0.01, ***P < 0.001

Bioinformatics analysis was performed to investigate the possible binding target of LRP11-AS1. The possible interacting miRNAs were predicted by LncRNASNP2 (http://bioinfo.life.hust.edu.cn/lncRNASNP#!/) and the miRNAs with binding energy less than -20 were shown in Additional file 1: Fig. S7. After searching the biological functions of these miRNAs, miR-149-3p, miR-338-3p and miR-4508 were selected for further study since they were reported to be involved in cell growth, migration or as a tumor suppressive miRNA in breast cancer [9, 15, 16]. Figure 2A showed that TNBC cell line MDA-MB-231 and MDA-MB-468 showed significantly lower expression of miR-149-3p compared to the mammary epithelial cell line MCF10A. Even though LRP11-AS1 was not upregulated in MCF7, the expression of miR-149-3p was low in this cell line, which may be due to the regulation of other upstream regulators. Knockdown of LRP11-AS1 in TNBC cells upregulated the expression of the tumor suppressive miR-149-3p (Fig. 2B). Moreover, overexpression of miR-149-3p in TNBC cells suppressed the expression of LRP11-AS1 (Fig. 2C). Knockdown of LRP11-AS1 did not regulate the expression of miR-338-3p nor miR-4508, which were not investigated further. Dual-luciferase reporter assay was performed to investigate the binding between LRP11-AS1 and miR-149-3p. Figure 2D exhibited the possible binding sequence between LRP11-AS1 and miR-149-3p predicted by bioinformatics analysis. Figure 2E and F indicated that miR-149-3p could bind to LRP11-AS1 at the predicted binding site. These results indicated that miR-149-3p was a target of LRP11-AS1 in TNBC cells.

### Overexpression of miR-149-3p inhibited the growth and metastasis of TNBC cells

**Fig. 3:**
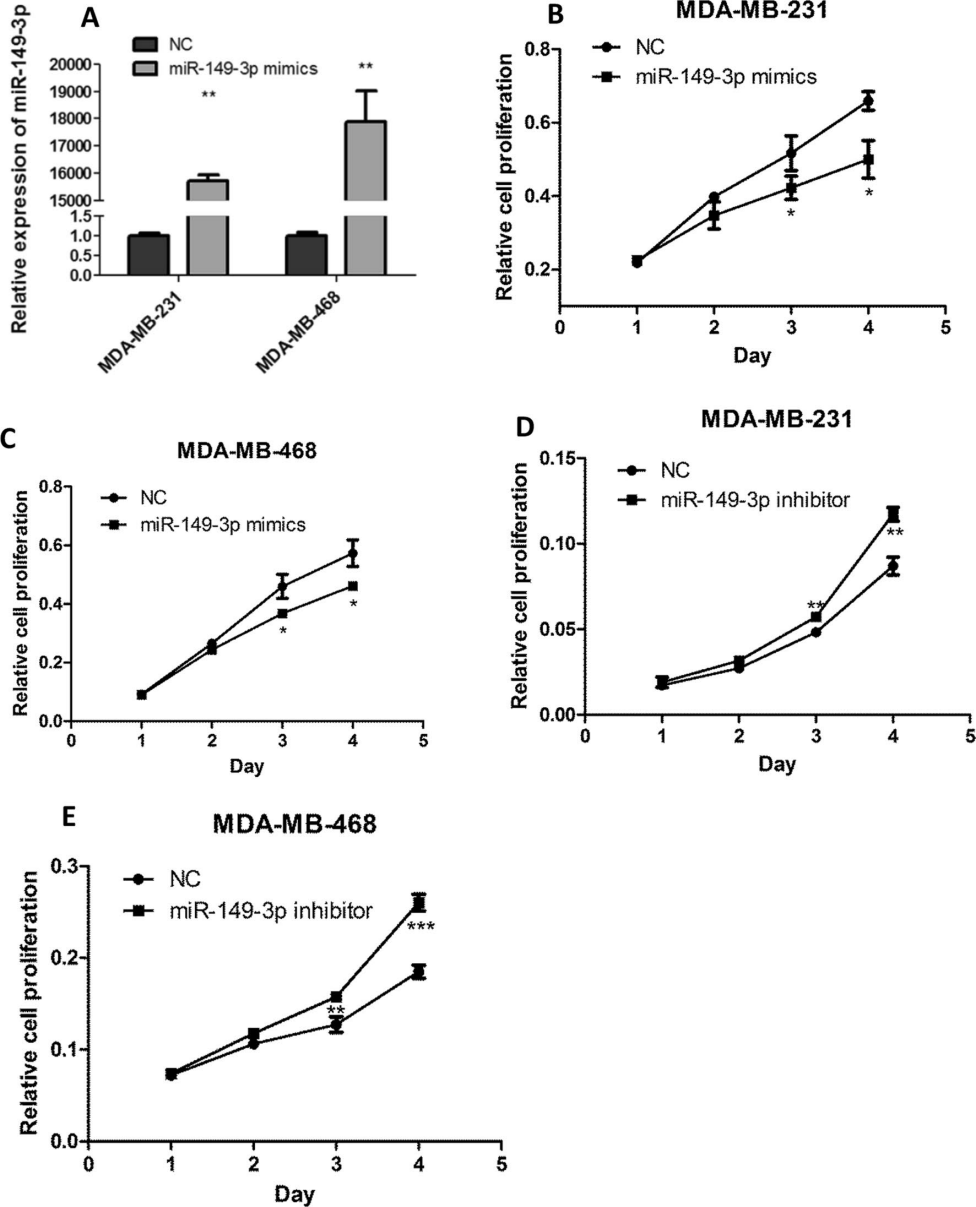

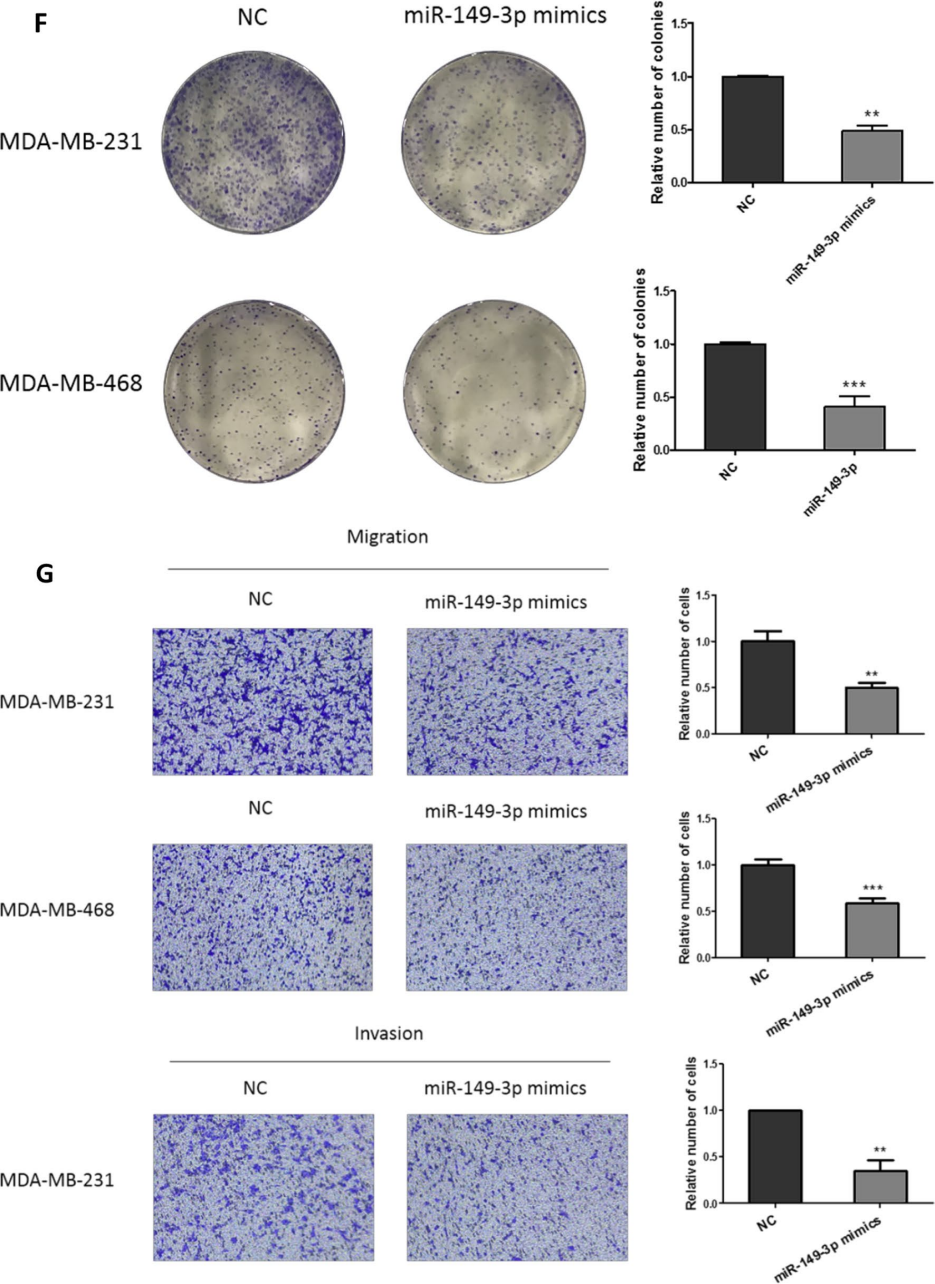
Overexpression of miR-149-3p inhibited the growth and metastasis of TNBC cells. **A** miR-149-3p was overexpressed in TNBC cells. The expression of miR-149-3p was evaluated by qPCR. **B**, **C** Overexpression of miR-149-3p inhibited the proliferation of TNBC cells. CCK-8 assay was performed to determine the proliferation of TNBC cells. **D**, **E** Inhibition of miR-149-3p promoted the proliferation of TNBC cells. **F** Overexpression of miR-149-3p abrogated the colony formation ability of TNBC cells. **G** Overexpression of miR-149-3p attenuated the migration and invasion ability of TNBC cells. The images were taken under ×4 magnification. Data were presented as mean ± SD. Statistic significant differences were indicated as *P < 0.05, **P < 0.01, ***P < 0.001

The effect of miR-149-3p on the growth and metastasis of TNBC was investigated. miR-149-3p was overexpressed in TNBC cells (Fig. 3A). Figure 3B and C showed that overexpression of miR-149-3p inhibited the proliferation of TNBC cells. Figure 3D and E showed that inhibition of miR-149-3p by inhibitors enhanced the proliferation of TNBC cells. Moreover, overexpression of miR-149-3p inhibited the colony formation (Fig. 3F), migration and invasion (Fig. 3G) of TNBC cells. And miR-149-3p inhibitor promoted the migration and invasion of TNBC cells (Additional file 1: Fig. S8).

### miR-149-3p inhibition reversed the anticancer effect of LRP11-AS1 deficiency in TNBC cells

**Fig. 4:**
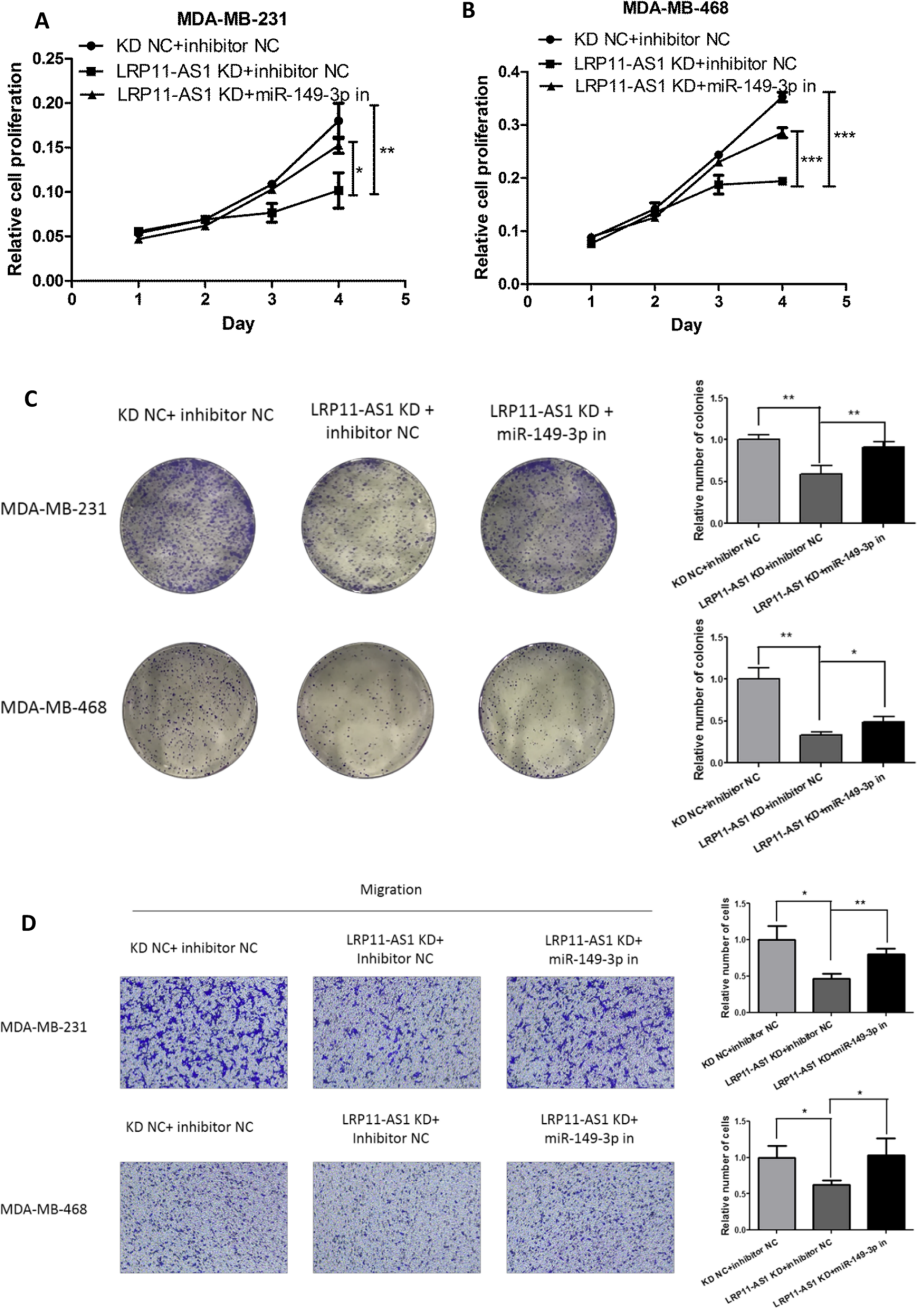

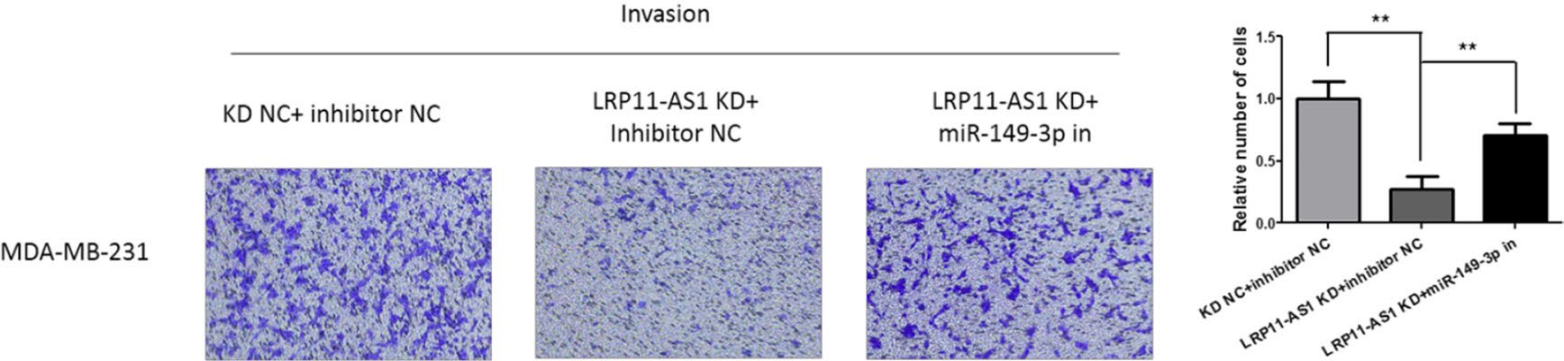
miR-149-3p inhibition reversed the anticancer effect of LRP11-AS1 deficiency in TNBC cells. **A**, **B** miR-149-3p inhibition partially reversed the inhibitory effect of LRP11-AS1 knockdown on the proliferation of TNBC. **C** miR-149-3p inhibition partially reversed the inhibitory effect of LRP11-AS1 knockdown on the colony formation of TNBC. **D** Inhibition of miR-149-3p partially restored the anticancer effect on the migration and invasion by LRP11-AS1 knockdown. The images were taken under ×4 magnification. Data were presented as mean ± SD. Statistic significant differences were indicated as *P < 0.05, **P < 0.01, ***P < 0.001

Rescue experiments were performed to investigate whether LRP11-AS1 promoted the growth and metastasis of TNBC cells by targeting miR-149-3p. Inhibition of miR-149-3p partially reversed the inhibitory effect of LRP11-AS1 deficiency on cell proliferation (Fig. 4A and B) and colony formation (Fig. 4C) in TNBC cells. Figure 4D exhibited that inhibition of miR-149-3p partially restored the inhibitory effect of LRP11-AS1 knockdown on the migration and invasion of TNBC cells. These results indicated that knockdown of LRP11-AS1 inhibited the growth and metastasis of TNBC cells by targeting miR-149-3p.

### NRP2 was a target of miR-149-3p and LRP11-AS1 regulated NRP2 through miR-149-3p

**Fig. 5:**
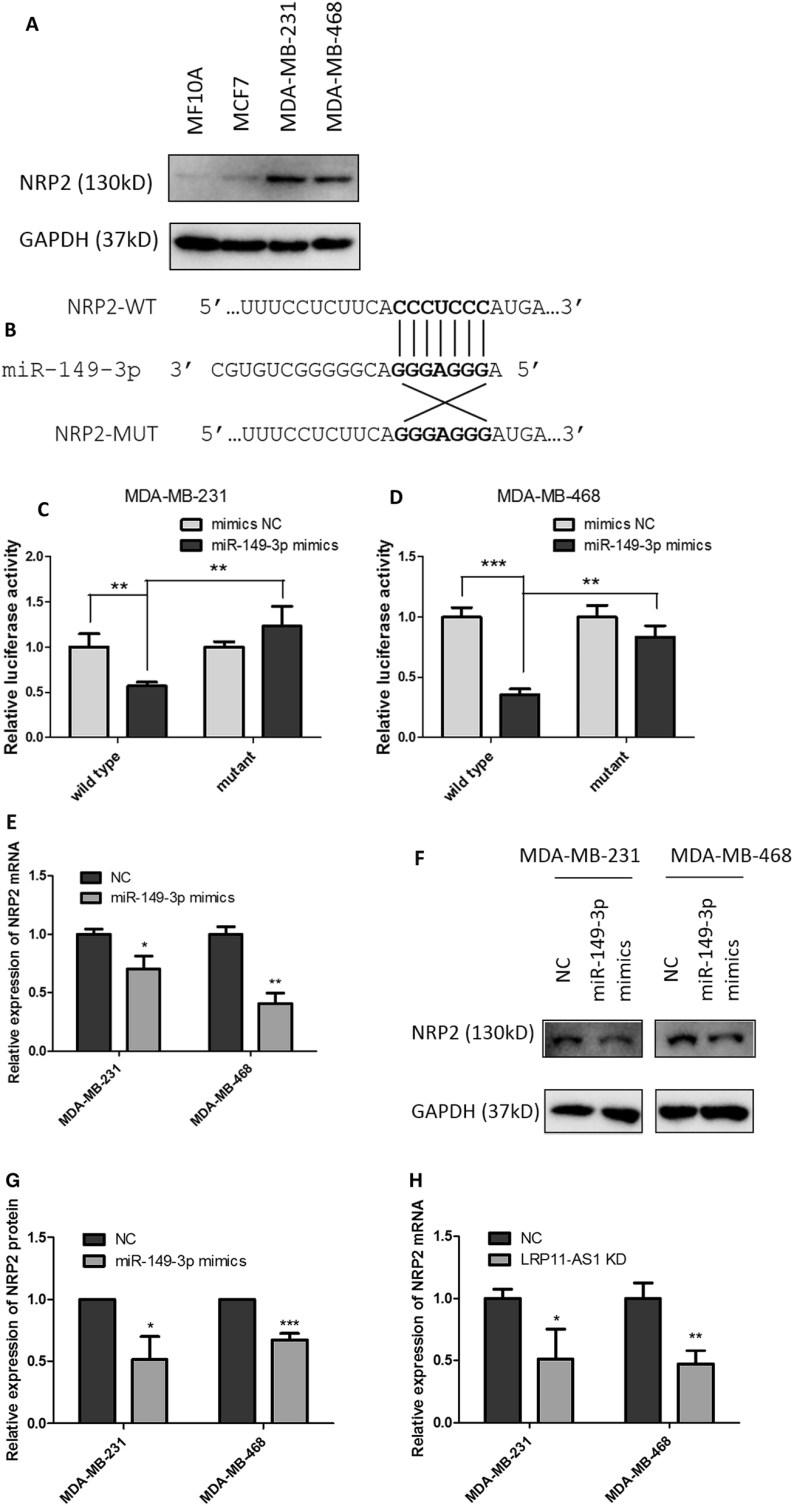

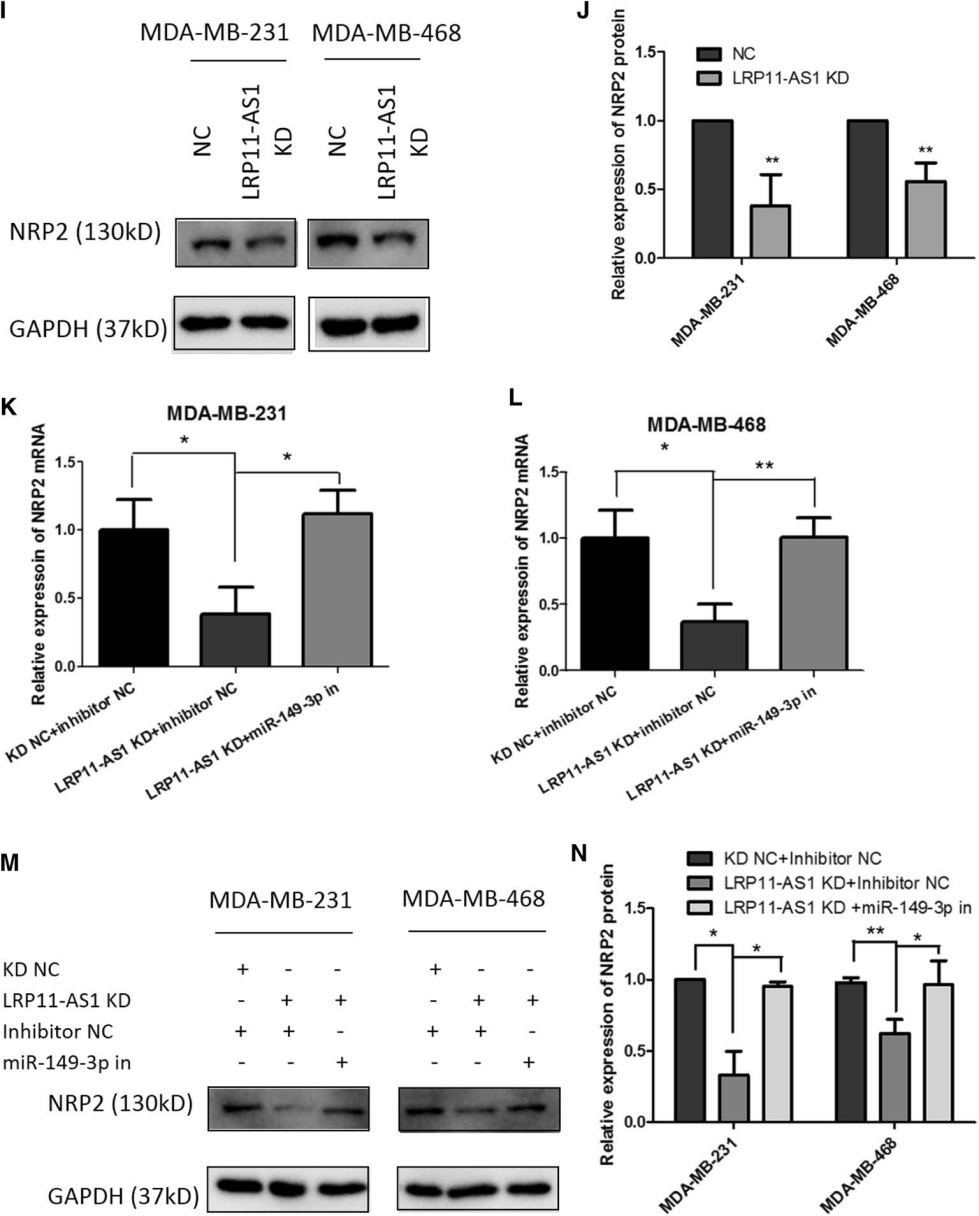
NRP2 was a target of miR-149-3p and LRP11-AS1 regulated NRP2 through miR-149-3p. **A** NRP2 was found to be overexpressed in TNBC cells compared to the non-TNBC cells and normal mammary epithelial cells. The expression of NRP2 was evaluated by western blot. **B** The predicted binding sequence between NRP2 and miR-149-3p. **C**, **D** Dual-luciferase reporter assay of the interaction between NRP2 and miR-149-3p. Cells were co-transfected with miRNA mimics and the dual-luciferase reporter plasmid. Luciferase activity was measured at 48 h post-transfection. **E** Overexpression of miR-149-3p downregulated the expression of NRP2 mRNA. The expression of NRP2 was evaluated by qPCR. **F**, **G** Overexpression of miR-149-3p downregulated the expression of NRP2 protein. The expression of NRP2 was evaluated by western blot. **H** Knockdown of LRP11-AS1 downregulated the expression of NRP2 mRNA. The expression of NRP2 was evaluated by qPCR. **I**, **J** Knockdown of LRP11-AS1 downregulated the expression of NRP2 protein. The expression of NRP2 was evaluated by western blot. **K**, **L** LRP11-AS1 regulated the expression of NRP2 mRNA through miR-149-3p. The expression of NRP2 was evaluated by qPCR. **M**, **N** LRP11-AS1 regulated the expression of NRP2 protein through miR-149-3p. The expression of NRP2 was evaluated by western blot. Data were presented as mean ± SD. Statistic significant differences were indicated as *P < 0.05, **P < 0.01, ***P < 0.001

The downstream targets of miR-149-3p were predicted using online bioinformatics analysis tool TargetScan (http://www.targetscan.org/vert_72/). The top 10 predicted targets were listed in Additional file 1: Fig. S9. The expression of the top 3 targets was measured after the overexpression of miR-149-3p. The expression of the 3 targets were not significantly affected. Five more targets of miR-149-3p were selected among the top 2000 predicted targets according to TargetScan. These genes were TP53, AKT2, MMP2, FOXC2 and NRP2, which were reported to be involved in the proliferation, migration or invasion of cancers. Among these 5 predicted targets, Neuropilin-2 (NRP2) was the only gene found to be regulated by miR-149-3p. Figure 5A showed the relative expression of NRP2 in different cell lines. TNBC cell line MDA-MB-231 and MDA-MB-468 showed higher expression of NRP2 compared to the non-TNBC cell line MCF7 and mammary epithelial cell line MCF10A. Figure 5B represented the possible binding site on the 3′-untranslated region (UTR) of NRP2 to miR-149-3p. Dual-luciferase reporter assay indicated the binding of miR-149-3p to NRP2 at the predicted binding site (Fig. 5C and D). Overexpression of miR-149-3p downregulated the mRNA (Fig. 5E) and protein (Fig. 5F and G) levels of NRP2 in TNBC cells. Silencing of LRP11-AS1 inhibited the mRNA (Fig. 5H) and protein (Fig. 5I and J) expression of NRP2 in TNBC cells. Rescue experiments indicated that inhibition of miR-149-3p restored the suppression of NRP2 by LRP11-AS1 knockdown in both of the transcriptional (Fig. 5K and L) and translational (Fig. 5M and 5N) levels. These results suggested that NRP2 was a target of miR-149-3p and NRP2 was regulated by LRP11-AS1 through miR-149-3p.

### Overexpression of NRP2 reversed the anticancer effect of LRP11-AS1 deficiency in TNBC cells

**Fig. 6:**
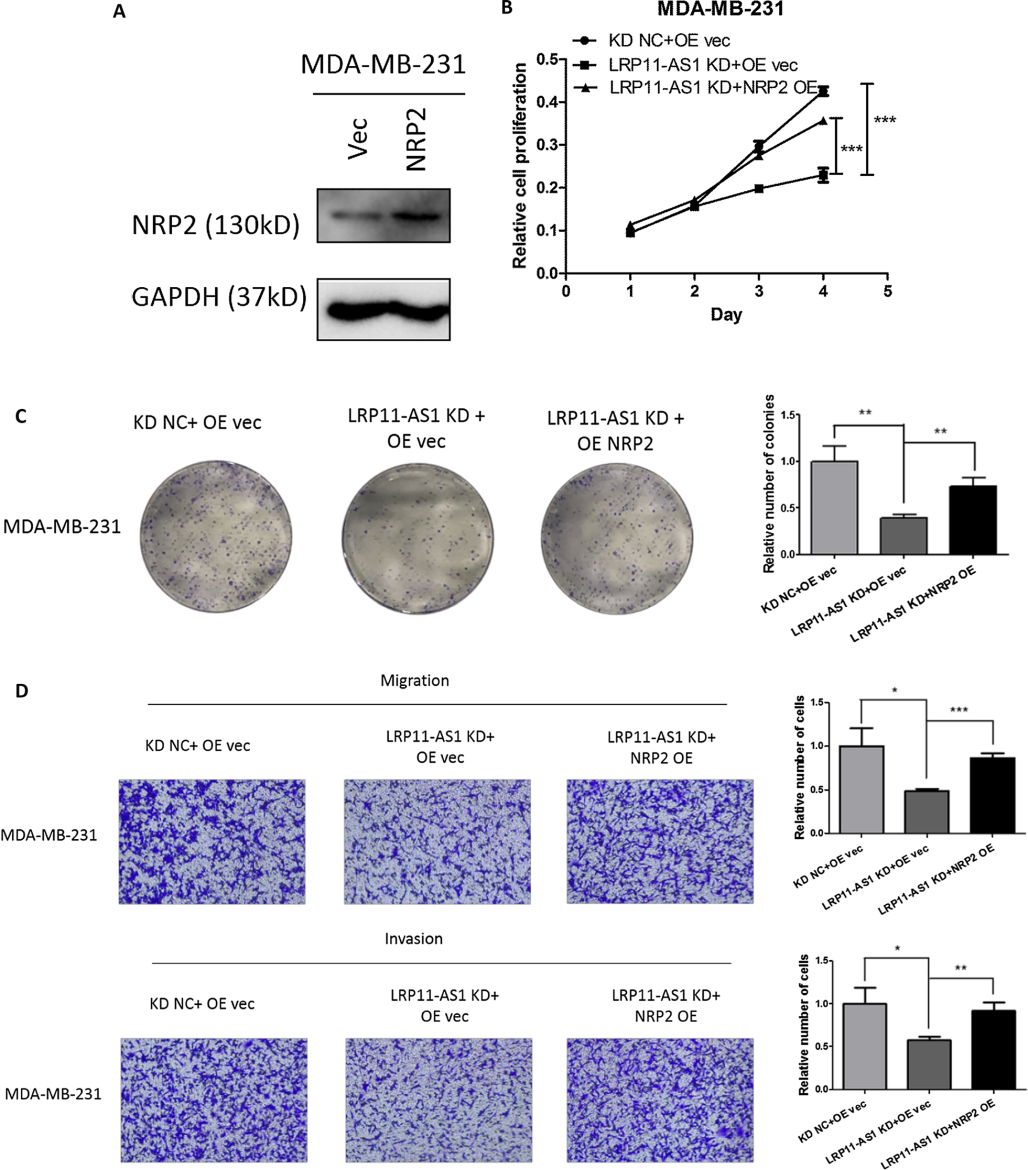
Overexpression of NRP2 reversed the anticancer effect of LRP11-AS1 deficiency in TNBC cells. **A** Overexpression of NRP2 in TNBC cells. MDA-MB-231 cells were transfected with NRP2 or vector control plasmid. The expression of NRP2 was evaluated by western blot. **B** Overexpression of NRP2 partially reversed the inhibitory effect of LRP11-AS1 deficiency on cell proliferation. CCK-8 assay was performed to determine the proliferation of TNBC cells. **C** Overexpression of NRP2 partially reversed the inhibition of the colony formation by LRP11-AS1 knockdown. **D** Overexpression of NRP2 partially reversed the inhibition of the migration and invasion by LRP11-AS1 knockdown. The images were taken under ×4 magnification. Data were presented as mean ± SD. Statistic significant differences were indicated as *P < 0.05, **P < 0.01, ***P < 0.001

Since NRP2 was determined to be the target of miR-149-3p and was regulated by LRP11-AS1, rescue experiments were performed by overexpressing NRP2 in TNBC cells. Figure 6A showed the overexpression of NRP2 in MDA-MB-231 cells. The overexpression of NRP2 in MDA-MB-468 was insignificant. Overexpression of NRP2 partially rescued the inhibitory effect of LRP11-AS1 deficiency on cell proliferation (Fig. 6B). Overexpression of NRP2 also partially rescued the inhibitory effect of LRP11-AS1 deficiency on the colony formation (Fig. 6C), migration and invasion (Fig. 6D) of TNBC cells. These results indicated that knockdown of LRP11-AS1 inhibited the growth and metastasis of TNBC cells by inhibiting NRP2. LRP11-AS1 that was overexpressed in TNBC cells promoted the growth and metastasis of TNBC cells by targeting the miR-149-3p/NRP2 axis.

## Discussion

Triple negative breast cancer (TNBC) is a very aggressive subtype of breast cancer characterized by a deficiency in the hormone receptors, which could serve as drug targets. Chemotherapy is the only recommended systemic treatment with inevitable resistance or relapse. In this study, lncRNA LRP11-AS1 was firstly reported to be overexpressed in TNBC cells compared to the non-TNBC cells and normal mammary epithelial cells. Functional studies suggested that silencing of LRP11-AS1 inhibited the growth and metastasis of breast cancers and induced cell cycle arrest.

Mechanistically, miR-149-3p was predicted to be the target of LRP11-AS1 by bioinformatics analysis. miR-149-3p was previously reported to be downregulated in breast cancer clinical samples [9, 17]. In our study, low expression of miR-149-3p was found in TNBC cell lines. Overexpression of miR-149-3p could inhibit the growth and metastasis of TNBC, which indicated the tumor suppressive role of miR-149-3p in TNBC cells. Dual-luciferase reporter assay proved the binding of LRP11-AS1 with miR-149-3p in the predicted binding site. More importantly, knockdown of LRP11-AS1 increased the expression of miR-149-3p and overexpression of miR-149-3p reduced the expression of LRP11-AS1. Rescue experiments showed that inhibition of miR-149-3p could partially rescue the anticancer effect of LRP11-AS1 deficiency in TNBC cells. These results indicated that LRP11-AS1 possibly served as the ceRNA to sponge miR-149-3p to promote the growth and metastasis of TNBC.

**Fig. 7:**
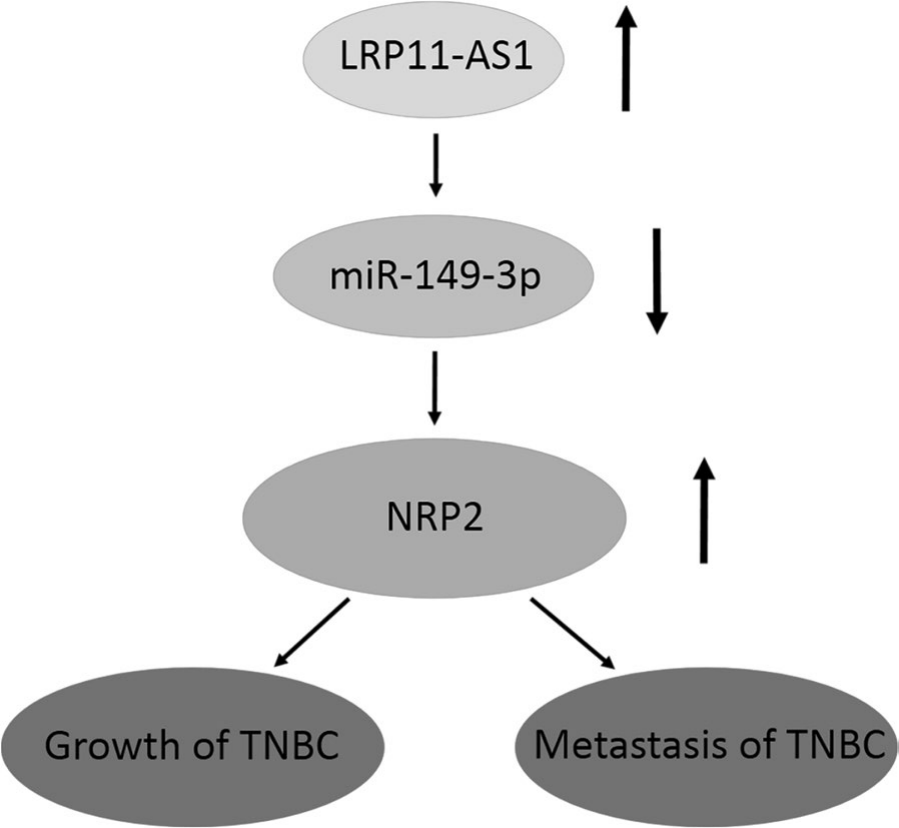
LRP11-AS1 promoted the growth and metastasis of TNBC cells by targeting the miR-149-3p/NRP2 axis. A graphic description of the mechanisms underlying the oncogenic role of LRP11-AS1 in TNBC cells. The overexpression of LRP11-AS1 in TNBC suppressed the expression of miR-149-3p and therefore, enhanced the expression of NRP2, which resulted in the promotion of the growth and metastasis of TNBC cells. LRP11-AS1 exhibited the malignant behavior in TNBC by sponging miR-149-3p and regulating the miR-149-3p/NRP2 axis

NRP2 was previously reported as a member of the membrane protein neuropilin family. Increased expression of NRP2 was found in invasive and TNBC and was correlated with lymph node metastasis [12, 14, 18, 19]. In our study, dual-luciferase reporter assay revealed the interaction between miR-149-3p and the 3′-UTR of NRP2. miR-149-3p mimics could inhibit the expression of NRP2 in TNBC cells. Moreover, silencing of LRP11-AS1 could also inhibit the expression of NRP2 in TNBC cells. Rescue experiments represented that inhibition of miR-149-3p could restore the inhibited expression of NRP2 by LRP11-AS1 knockdown, indicating that LRP11-AS1 regulated NRP2 expression through miR-149-3p. Functionally, overexpression of NRP2 could partially reverse the anticancer effect of LRP11-AS1 deficiency on the proliferation, migration and invasion of TNBC cells. Figure 7 represented a graphic description of the possible mechanisms of the oncogenic role of LRP11-AS1 played in TNBC. In our results, bioinformatics analysis predicted the possible binding sequence between LRP11-AS1 and miR-149-3p (Fig. 2D) and knockdown of LRP11-AS1 could promote the expression of miR-149-3p (Fig. 2B). The overexpression of LRP11-AS1 in TNBC lead to the suppression of miR-149-3p and the upregulation of NRP2, which resulted in the promotion of the growth and metastasis of TNBC cells. LRP11-AS1 exhibited the tumorigenic effect in TNBC by sponging miR-149-3p and targeting the miR-149-3p/NRP2 axis. LRP11-AS1 could serve as a potential biomarker in the diagnosis and treatment of TNBC. Limitations still exist in this study, even though TNBC cell lines overexpress LRP11-AS1, the expression of this lncRNA in TNBC clinical samples needs investigation. After validating the function of LRP11-AS1 in vitro, the role and mechanism of this novel lncRNA could be further studied in vivo in the future research.

## Conclusions

LncRNA LRP11-AS1, which was overexpressed in TNBC cells could promote the growth and metastasis of TNBC cells by working as a ceRNA to sponge miR-149-3p and regulating the miR-149-3p/NRP2 axis, indicating LRP11-AS1 as a potential biomarker in the diagnosis and treatment of TNBC.

## Supplementary Information

**Additional file 1.** Additional figures.

## Data Availability

The datasets used and/or analyzed during the current study are available from the corresponding author on reasonable request.

## Abbreviations

TNBC: Triple negative breast cancer
lncRNA: Long noncoding RNA
ceRNA: Competing endogenous RNA
LRP11: Low-density lipoprotein (LDL) receptor related protein 11
LRP11-AS1: LRP11 antisense RNA 1
NRP2: Neuropilin-2
miRNAs: MicroRNAs
3′-UTR: 3′-Untranslated region

## References

[1] Sung H, Ferlay J, Siegel RL, Laversanne M, Soerjomataram I, Jemal A, Bray F (2021). Global cancer statistics 2020: GLOBOCAN estimates of incidence and mortality worldwide for 36 cancers in 185 countries. Cancer J Clin.

[2] McDonald ES, Clark AS, Tchou J, Zhang P, Freedman GM (2016). Clinical diagnosis and management of breast cancer. J Nucl Med.

[3] Medina MA, Oza G, Sharma A, Arriaga LG, Hernández Hernández JM, Rotello VM, Ramirez JT (2020). Triple-negative breast cancer: a review of conventional and advanced therapeutic strategies. Int J Environ Res Public Health.

[4] Kumar P, Aggarwal R (2016). An overview of triple-negative breast cancer. Arch Gynecol Obstet.

[5] Peng WX, Koirala P, Mo YY (2017). LncRNA-mediated regulation of cell signaling in cancer. Oncogene.

[6] Dykes IM, Emanueli C (2017). Transcriptional and post-transcriptional gene regulation by long non-coding RNA. Genomics Proteomics Bioinform.

[7] Sathish Kumar B, Kumar A, Singh J, Hasanain M, Singh A, Fatima K, Yadav DK, Shukla V, Luqman S, Khan F (2014). Synthesis of 2-alkoxy and 2-benzyloxy analogues of estradiol as anti-breast cancer agents through microtubule stabilization. Eur J Med Chem.

[8] Goedert L, Plaça JR, Fuziwara CS, Machado MCR, Plaça DR, Almeida PP, Sanches TP, Santos JF, Corveloni AC, Pereira IEG (2017). Identification of long noncoding RNAs deregulated in papillary thyroid cancer and correlated with BRAFV600E mutation by bioinformatics integrative analysis. Sci Rep.

[9] Dong Y, Chang C, Liu J, Qiang J (2017). Targeting of GIT1 by miR-149* in breast cancer suppresses cell proliferation and metastasis in vitro and tumor growth in vivo. Onco Targets Ther.

[10] Srivastava A, Fatima K, Fatima E, Singh A, Singh A, Shukla A, Luqman S, Shanker K, Chanda D, Khan F (2020). Fluorinated benzylidene indanone exhibits antiproliferative activity through modulation of microtubule dynamics and antiangiogenic activity. Eur J Pharm Sci.

[11] Rizzolio S, Tamagnone L (2011). Multifaceted role of neuropilins in cancer. Curr Med Chem.

[12] Zhao M, Zhang M, Tao Z, Cao J, Wang L, Hu X (2020). miR-331-3p suppresses cell proliferation in TNBC cells by downregulating NRP2. Technol Cancer Res Treat.

[13] Sathish Kumar B, Singh A, Kumar A, Singh J, Hasanain M, Singh A, Masood N, Yadav DK, Konwar R, Mitra K (2014). Synthesis of neolignans as microtubule stabilisers. Bioorg Med Chem.

[14] Yasuoka H, Kodama R, Tsujimoto M, Yoshidome K, Akamatsu H, Nakahara M, Inagaki M, Sanke T, Nakamura Y (2009). Neuropilin-2 expression in breast cancer: correlation with lymph node metastasis, poor prognosis, and regulation of CXCR4 expression. BMC Cancer.

[15] He J, Wang J, Li S, Li T, Chen K, Zhang S (2020). Hypoxia-inhibited miR-338-3p suppresses breast cancer progression by directly targeting ZEB2. Cancer Sci.

[16] Boo L, Ho WY, Ali NM, Yeap SK, Ky H, Chan KG, Yin WF, Satharasinghe DA, Liew WC, Tan SW (2016). MiRNA transcriptome profiling of spheroid-enriched cells with cancer stem cell properties in human breast MCF-7 cell line. Int J Biol Sci.

[17] Kumar BS, Ravi K, Verma AK, Fatima K, Hasanain M, Singh A, Sarkar J, Luqman S, Chanda D, Negi AS (2017). Synthesis of pharmacologically important naphthoquinones and anticancer activity of 2-benzyllawsone through DNA topoisomerase-II inhibition. Bioorg Med Chem.

[18] Khwaja S, Fatima K, Hasanain M, Behera C, Kour A, Singh A, Luqman S, Sarkar J, Chanda D, Shanker K (2018). Antiproliferative efficacy of curcumin mimics through microtubule destabilization. Eur J Med Chem.

[19] Singh A, Fatima K, Singh A, Behl A, Mintoo MJ, Hasanain M, Ashraf R, Luqman S, Shanker K, Mondhe DM (2015). Anticancer activity and toxicity profiles of 2-benzylidene indanone lead molecule. Eur J Pharm Sci.

