# Supplementary material for: LRP11-AS1 promotes the proliferation and migration of triple negative breast cancer cells via the miR-149-3p/NRP2 axis
Source: Cancer Cell Int. 2022 Mar 12;22:116. doi: 10.1186/s12935-022-02536-8 (PMC8917722; doi:10.1186/s12935-022-02536-8)
Supplement: Supplementary file 1 — Additional file 1. Additional figures. [file 12935_2022_2536_MOESM1_ESM.docx]

S1





Figure S1 Knockdown of LRP11-AS1 inhibited the proliferation of TNBC cells. CCK-8 assay was performed to determine the proliferation of TNBC cells. Data in the supplementary files were presented as mean±SD. Statistic significant differences were indicated as *P < 0.05, **P < 0.01, ***P<0.001.

S2





Figure S2 Overexpression of LRP11-AS1 promoted the proliferation of TNBC cells.

S3


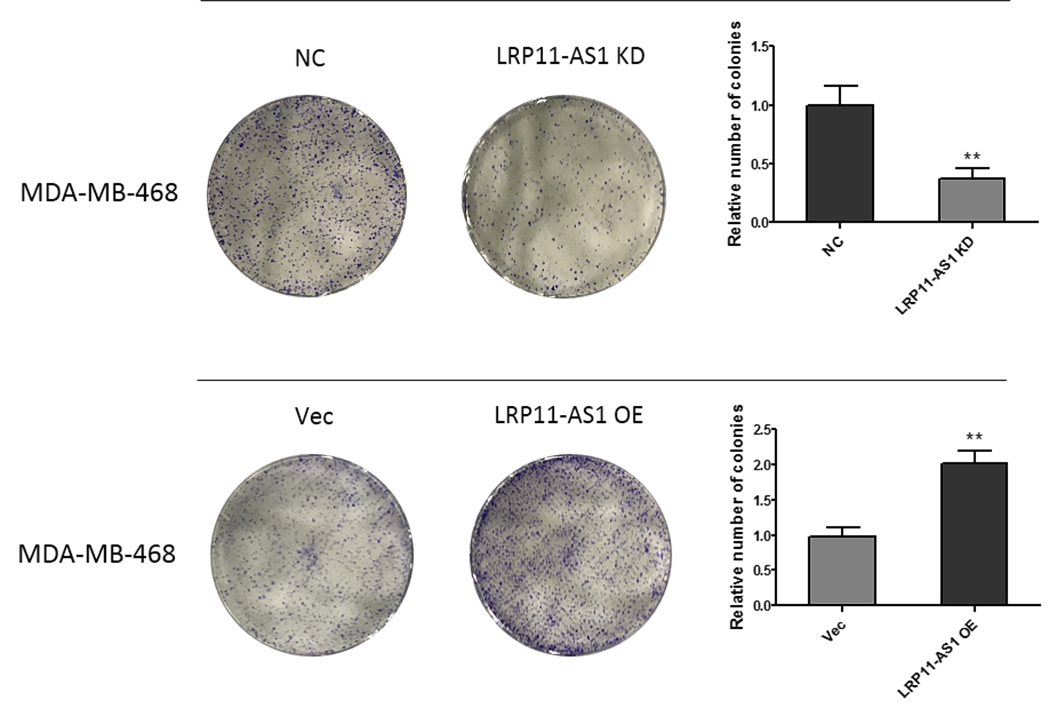


Figure S3 F, Colony formation assay of TNBC cells silenced of LRP11-AS1 or overexpressed with LRP11-AS1

S4


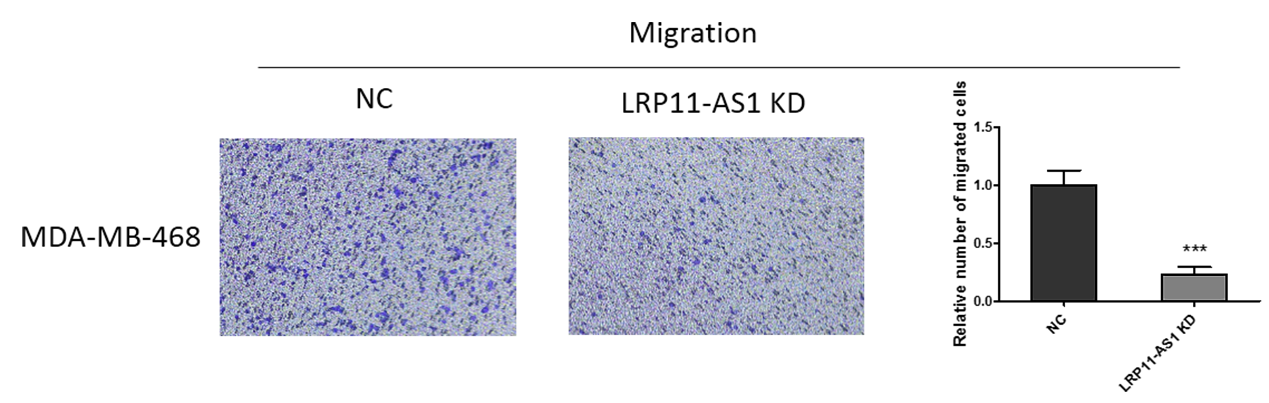


Figure S4 Silencing of LRP11-AS1 inhibited the migration of TNBC cells. The images were taken under 4X magnification.

S5


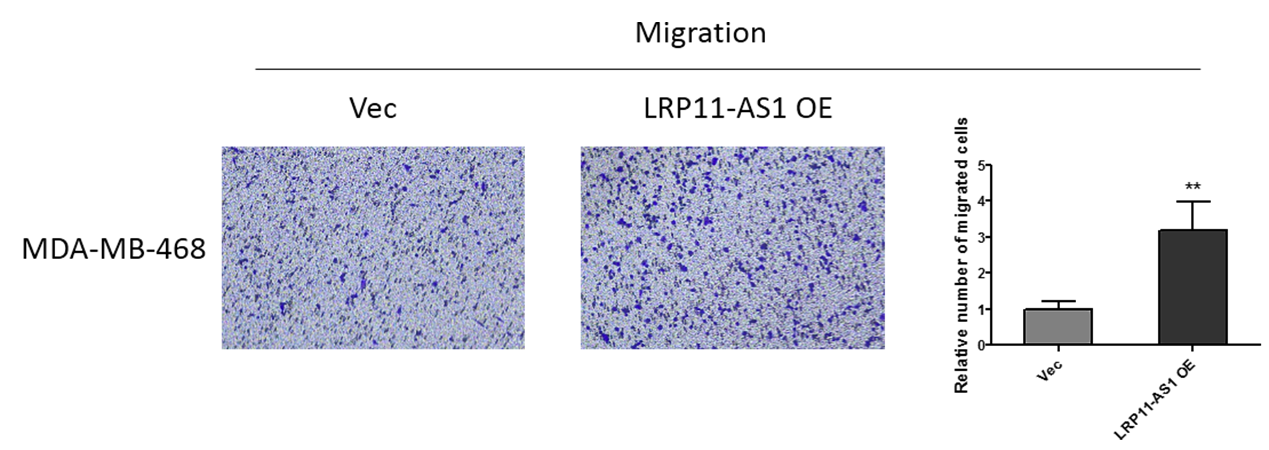


Figure S5 Overexpression of LRP11-AS1 enhanced the migration of TNBC cells. The images were taken under 4X magnification.

S6


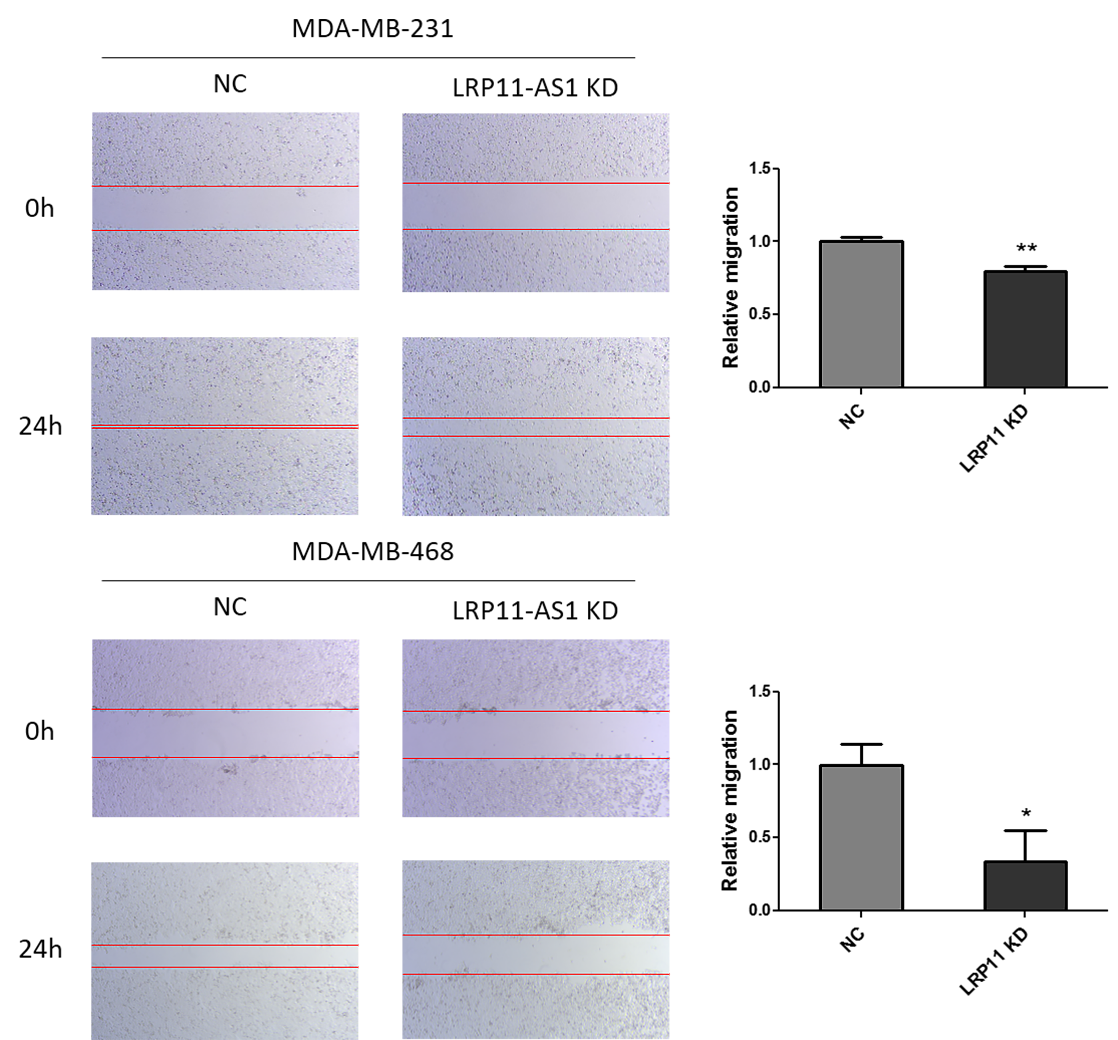


Figure S6 Silencing of LRP11-AS1 inhibited the wound healing migration of TNBC cells. The images were taken under 4X magnification.

S7


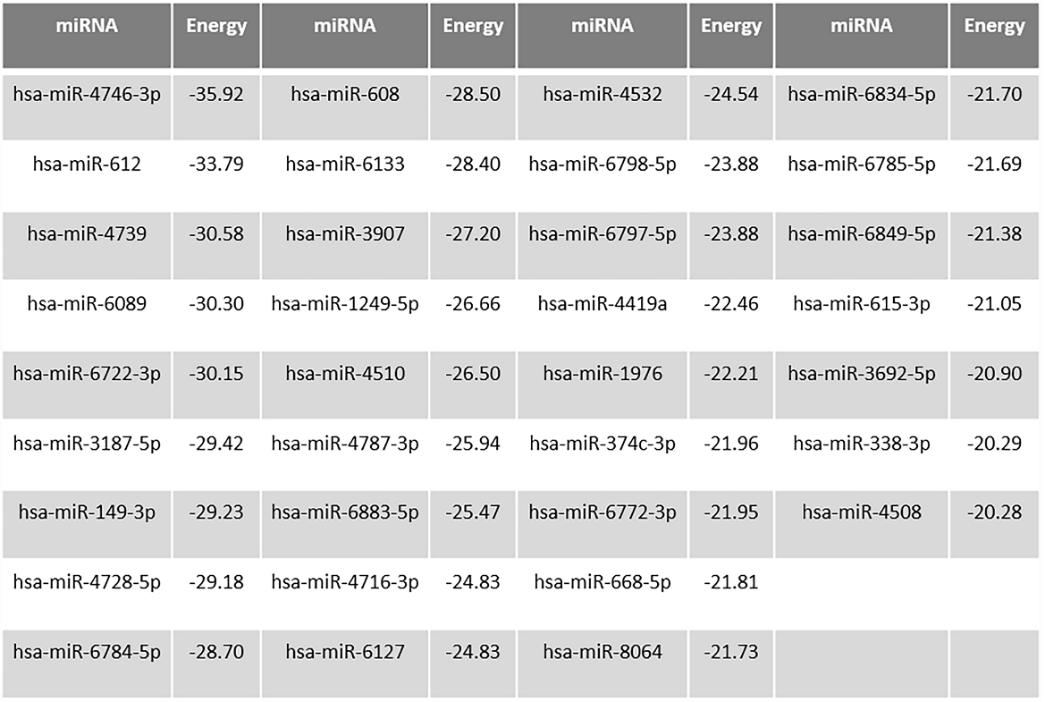


Figure S7 Top possible interacting miRNAs of LRP11-AS1 predicted by LncRNASNP2. miRNAs with binding energy less than -20 were shown.

S8


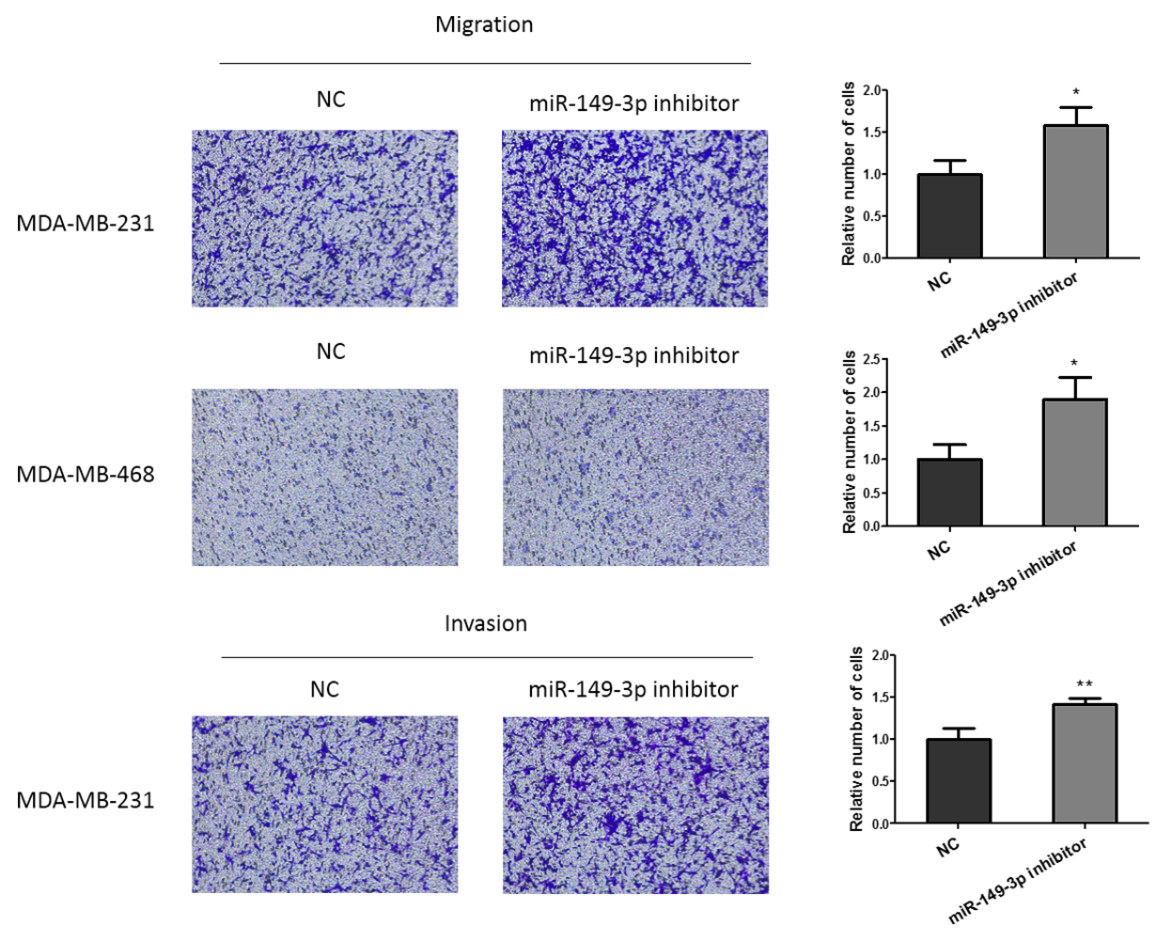


Figure S8 Inhibition of miR-149-3p enhanced the migration and invasion of TNBC cells. The images were taken under 4X magnification.S9


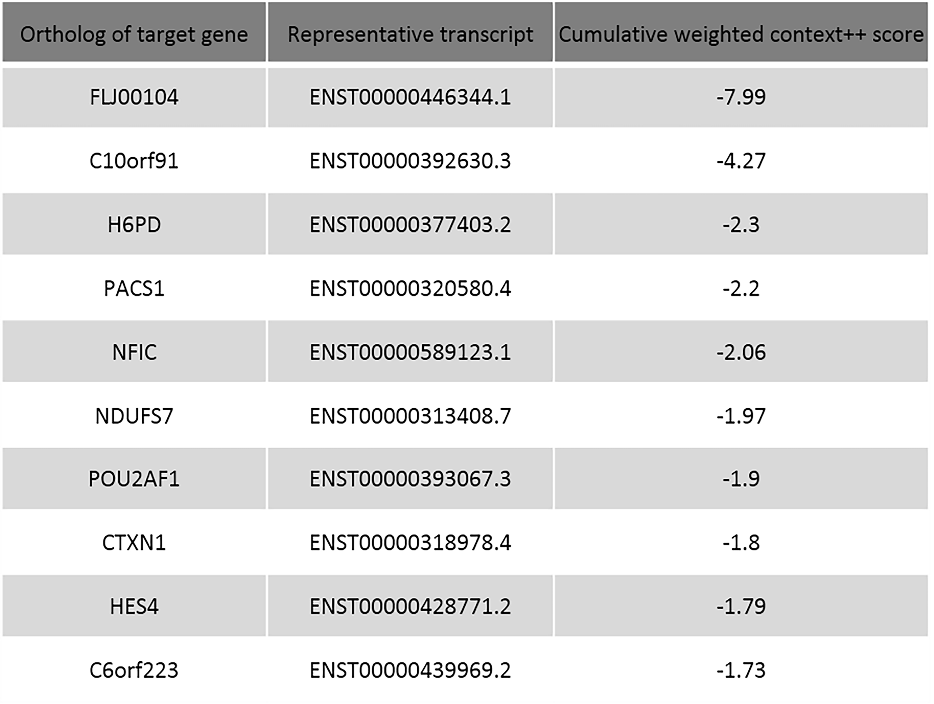


Figure S9 The possible targets of miR-149-3p predicted by TargetScan. The top 10 predicted targets were listed.
